# Lead and Neuroprotection by Iron in ADHD

**DOI:** 10.1289/ehp.10304

**Published:** 2007-08

**Authors:** Eric Konofal, Samuele Cortese

**Affiliations:** Child Psychopathology Unit, University Hospital Robert Debré, Paris, France, E-mail: eric.konofal@rdb.aphp.fr

We read with special interest the article by Braun et al. (2006). In this large survey, the authors concluded that prenatal exposure to tobacco and environmental lead are risk factors for attention deficit hyperactivity disorder (ADHD).

We would like to focus on the potential neuroprotective role of iron against the deleterious effect of lead on the development of ADHD symptoms.

Although the mechanisms underlying ADHD remain unclear, both genetic and environmental factors have been implicated. In a recent review on the implication of the dopaminergic system in the etiology of ADHD, Swanson et al. (2007) highlighted the importance of environmental risk factors as possible etiologies of dopamine deficit. Among these environmental factors, Swanson et al. (2007) cited the effects of lead exposure (at levels < 10 μg/dL) on ADHD-related behaviors and ADHD diagnosis.

Lead in the central nervous system may contribute to dopaminergic dysfunction inducing alteration of dopamine release and dopamine receptor density (Gedeon et al. 2001; Lidsky et al. 2003). Moreover, lead may disrupt the structure of the blood–brain barrier function essential for brain integrity (Dyatlov et al. 1998). Interestingly, Wang et al. (2007) recently reported that iron supplementation protects the integrity of the blood–brain barrier against lead insults. On the other hand, iron deficiency could increase the toxic effect of lead, suggesting a potent neuroprotective effect of iron supplementation on dopaminergic dysfunction due to lead exposure (Wright 1999; Wright et al. 2003)

In a controlled comparison group study, we (Konofal et al. 2004) showed that iron deficiency was correlated to ADHD symptoms severity, hypothesizing that iron supplementation may improve symptoms of ADHD in those subjects with low ferritin levels.

Given that lead exposure may contribute to ADHD and iron deficiency may exacerbate deleterious effects caused by lead, we recommend systematically seeking for iron deficiency in children with ADHD. We also think that controlled studies assessing the potential effectiveness of iron supplementation on ADHD symptoms should be encouraged. Such studies could aid the understanding of the complex pathophysiology underlying ADHD and provide effective therapeutic strategies for this disorder.

## References

[b1-ehp0115-a0398b] Braun JM, Kahn RS, Froehlich T, Auinger P, Lanphear BP (2006). Exposures to environmental toxicants and attention deficit hyperactivity disorder in U.S. children. Environ Health Perspect.

[b2-ehp0115-a0398b] Dyatlov VA, Platoshin AV, Lawrence DA, Carpenter DO (1998). Lead potentiates cytokine- and glutamate-mediated increases in permeability of the blood-brain barrier. Neurotoxicology.

[b3-ehp0115-a0398b] Gedeon Y, Ramesh GT, Wellman PJ, Jadhav AL (2001). Changes in mesocorticolimbic dopamine and D1/D2 receptor levels after low level lead exposure: a time course study. Toxicol Lett.

[b4-ehp0115-a0398b] Konofal E, Lecendreux M, Arnulf I, Mouren MC (2004). Iron deficiency in children with attention-deficit/hyperactivity disorder. Arch Pediatr Adolesc Med.

[b5-ehp0115-a0398b] Lidsky TI, Schneider JS (2003). Lead neurotoxicity in children: basic mechanisms and clinical correlates. Brain.

[b6-ehp0115-a0398b] Swanson JM, Kinsbourne M, Nigg J, Lanphear B, Stefanatos GA, Volkow N (2007). Etiologic subtypes of attention-deficit/hyperactivity disorder: brain imaging, molecular genetic and environmental factors and the dopamine hypothesis. Neuropsychol Rev.

[b7-ehp0115-a0398b] Wang Q, Luo W, Zheng W, Liu Y, Xu H, Zheng G (2007). Iron supplement prevents lead-induced disruption of the blood-brain barrier during rat development. Toxicol Appl Pharmacol.

[b8-ehp0115-a0398b] Wright RO (1999). The role of iron therapy in childhood plumbism. Curr Opin Pediatr.

[b9-ehp0115-a0398b] Wright RO, Tsaih SW, Schwartz J, Wright RJ, Hu H (2003). Association between iron deficiency and blood lead level in a longitudinal analysis of children followed in an urban primary care clinic. J Pediatrics.

